# Effects of Sand Surface Plyometric and Sprint Training on Physical and Technical Skill Performance in Beach Handball Players

**DOI:** 10.5114/jhk/169519

**Published:** 2023-10-11

**Authors:** Eduardo Sáez de Villarreal, Pedro Bago Rascón, Manuel Ortega Becerra, Julio Calleja-González, Pedro E. Alcaraz, Javier Feito-Blanco, Rodrigo Ramirez-Campillo

**Affiliations:** 1 Physical Performance Sports Research Center (PPSRC), Universidad Pablo de Olavide, Sevilla, Spain.; 2 Department of Physical Education and Sports, Faculty of Education and Sport, University of the Basque Country, Vitoria-Gasteiz, Spain.; 3 UCAM Research Center for High-Performance Sport, Catholic University San Antonio, Murcia, Spain.; 4 University of Oviedo, Consejería de Educación de Asturias, Oviedo, Spain.; 5 Exercise and Rehabilitation Sciences Institute, School of Physical Therapy, Faculty of Rehabilitation Sciences, Universidad Andres Bello, Santiago, Chile.

**Keywords:** physical conditioning, muscle strength, resistance training, team sports, musculoskeletal and neural physiological phenomena

## Abstract

This study compared the effects of a 6-week combined plyometric and sprint-training program on the sand to regular preseason training, on the athletic performance and technical actions of beach handball (BH) players. Athletes were randomly assigned either to the control (CG, n = 12; BH training only) or the experimental group (EG, n = 12; plyometric + sprint + BH training). Assessments conducted before and after the training period included a squat jump, a countermovement jump, the Abalakov jump, a 15-m sprint, a modified Course-Navette endurance test, and four sport-specific BH throwing speed tests: a standing penalty throw, a 3-step running throw, a jump throw, and a 360º jump throw. The training intervention enhanced all athletic performance measures (all, p < 0.05). In contrast, the only improvement in the CG included endurance performance (p< 0.05). Significant time-group differences were noted in favor of the EG compared to the CG (p< 0.05) in the squat jump, the countermovement jump, the Abalakov jump, the jump throw velocity and 360º jump throw velocity. In conclusion, compared to BH regular training, 6 weeks of sand surface preseason plyometric and sprint training combined with regular BH training induced greater improvements in athletic performance and specific skills in BH players.

## Introduction

Beach handball (BH) is played professionally worldwide. Despite the increasing professionalization, there is a paucity of research data concerning training interventions with most published studies involving court handball players. Additionally, coaches may adopt a conservative attitude toward training for BH, which limits the opportunities to obtain useful research data. Therefore, scientific interventions related to the training optimization process in BH should be a priority.

BH is a heterogeneous high-intensity sport with a variety of physiological demands involving displacements, throws and actions distributed intermittently throughout the game, with long periods of low-intensity activity, interspersed by short bursts of high-intensity activity (Pueo et al., 2017). As a result, players are exposed to both high- and low-level work rates which demand appropriate speed, acceleration ability, strength and power (Lara Cobos, 2011). High-speed actions (~4.4% of the total distance covered in BH matches) (Pueo et al., 2017) are decisive for winning possession of the ball and scoring goals (Sibila et al., 2004).

Although traditional strength training may increase force (Hermassi et al., 2011), high velocity and high rate of force development training may be required, such as plyometric exercises (Van Mujien et al., 1991). Plyometric training (PT) provides the required stimuli to enhance explosive contractions (i.e., a high rate of force development) (Sáez de Villarreal et al., 2010). Numerous studies have reported that PT alone can induce muscular performance benefits in lower limb muscles that also contribute to power development and represent a significant advantage of this type of training (Asadi et al., 2015; Markovic, 2007). Furthermore, a combination of PT with sprints may offer further advantages for team sport players (Aloui et al., 2022), although its effects on BH need confirmation.

An important consideration regarding the effects of PT and related sprint actions on muscle performance is the nature of the training surface. Water-based PT may reduce muscle soreness compared to PT on a firm surface, but with similar improvements in physical fitness (e.g., sprinting, jumping) (Arazi et al., 2012; Impellizzeri et al., 2007; Luberecka, 2022; Martel et al., 2005; Robinson et al., 2004). Lower soreness after aquatic versus land plyometric training may be related to the lower strain on the muscle-skeletal system in the later environmental condition (Robinson et al., 2004). Similarly, as a sand surface is associated with a greater degree of shock absorption and lower stress to soft tissue and bones on the lower limbs during plyometric exercise (Barrett et al., 1998; Boyanmis and Akin, 2022), less muscle soreness is observed after similar plyometric activity on a sand surface compared to a firm, wooden surface (Miyama and Nosaka, 2004).

PT on a sand surface can play a key role in shock absorption and reduce the stress on bones and tissue (Bishop, 2003). However, the friction and instability of sand can induce negative effects on the stretch-shortening-cycle (SSC), decreases in myotatic reflex, degradation of elastic energy potentiating and increases in the amortization phase, thus reducing the SSC potentiation (Giatsis et al., 2004; Impellizzeri et al., 2008). A recent meta-analysis (Pereira et al., 2021) demonstrated that sand surfaces were as efficient as hard surfaces to improve sprint and jump capacities in team-sport players. Moreover, although it was not part of the meta-analysis, some of the included studies also showed positive effects of sand training on COD and sprint ability (Binnie et al., 2014; Hammami et al., 2020; .Jastrzebskiet al., 2014; Pereira et al., 2022). From a neuromechanical perspective, the enhanced performance induced by plyometric sand training seems to be related to higher motor unit recruitment in the muscle groups involved in explosive movements (Pereira et al., 2023).

Although several studies have explored the effects of PT on land and sand surfaces, there is little information concerning the effectiveness of PT on sand in BH players. It would be relevant to examine the adaptability of plyometric and sprint training on the sand surface, given that some studies in the scientific literature have shown that sand can be one of the best surfaces for performing plyometrics and have demonstrated the efficiency of sand training in improving physical performance (Ahmadi et al., 2021; Pereira et al., 2021; Ramírez-Campillo et al., 2020). The low number of controlled studies of high methodological quality with elite athletes and the heterogeneous approaches of these studies preclude more robust conclusions on this issue.

Therefore, the study’s aim was to compare the effects of a sand surface plyometric and sprint-training intervention to a regular BH-training program on jumping, throwing, endurance and sprinting performance in BH players. We hypothesized greater physical fitness improvements after sand surface plyometric and sprint training compared to regular BH training.

## Methods

This study was designed to examine the effects of a PT + 15-m sprint training program, combined with regular BH practice, on athletes’ jumping, sprinting, endurance and throwing velocity performance, compared to regular BH training only. This study was conducted during 6 weeks (18 sessions) during the pre-season with BH players and involved low-volume and moderate-intensity training sessions. The distribution of the experimental and control groups was randomized (control group (CG), n = 12, only performed BH training; experimental group (EG), n = 12; BH training + plyometric + sprint training).

Physical performance was assessed before (pre) and after (post) the 6-week training period using a battery of tests as follows: i) 15-m sprint time with pre-activation (5 jumps and 5 s of skipping); ii) 15-m sprint time without pre-activation; iii) squat jump; iv) countermovement jump; v) Abalakov jump; vi) modified Course-Navette endurance test; vii) handball standing (penalty) throwing speed; viii) handball 3-step running throw speed; ix) handball jump throw speed; x) handball 360º jump throw speed.

All training sessions were supervised and performed during the afternoon (lasted 40 min) (before BH training), outdoors (on a BH court) with the temperature (28⁰C), wind (12 km/h), humidity (50%) and surface (sand) controlled. Participants maintained their usual dietary habits and normal BH training during the study.

### Participants

A group of 24 BH players from the Dos Hermanas BH Club, Sevilla, Spain, which is a Spanish first national division club, aged between 18 and 25 years (basic descriptive characteristics in Table 1) were recruited. Although with sporadic involvement in strength and power exercises (as part of the regular BH training routine), athletes had no systematic background in strength and power training. Athletes did not participate in training sessions or matches outside those scheduled for this study. All the participants signed an informed consent document. The study was conducted in accordance with the Declaration of Helsinki II and the study protocol was approved by the University Pablo de Olavide Ethics Committee.

**Table 1:** Participants’ descriptive characteristics.

	Age (y)	Height (cm)	Body mass (kg)	Experience* (y)	Tournaments** (n)	Matches**
EG (n = 12)	23.0 ± 4.9	180.9 ± 6.0	79.1 ± 8.3	5.3 ± 3.2	5.3 ± 1.9	29.4 ± 13.4
CG (n = 12)	24.3 ± 2.1	181.2 ± 4.4	81.2 ± 5.2	5.4 ± 2.4	5.7 ± 1.1	27.7 ± 15.1

* : experience as competitive beach handball players; **: number of beach handball tournaments or matches, completed during the year before enrollment in the study; EG: experimental group; CG: control group

### Testing Procedures

Players were familiarized with the tests two weeks before starting the training program. All tests were conducted over two days (Tuesday and Thursday). On day 1, players performed anthropometric measurements (body mass and height), vertical jump tests (cm), and the modified Course-Navette endurance test (min). On day 2, players performed the 15-m sprint test (s) and throwing speed tests (km/h). A standardized warm-up was performed before testing,and consisted of 7-min running at 9 km•h^−1^ followed by 5 min of joint mobilization exercises and active stretching, as well as 7 min of a specific BH warm-up (sprints, jumps). Sufficient rest was allowed between tests.

#### Vertical Jump Tests

An electronic contact platform (Ergo Jump Plus Bosco System^®^, Muscle Lab. V7. 18, Langesund, Norway) was used to measure the squat jump (SJ), countermovement jump (CMJ) and Abalakov jump test performance, in order to maximize the stretch-shortening cycle activity and to assess explosive strength of the lower limbs. The protocol for the tests has been previously reported (Ramírez-Campillo et al., 2013, 2019). The two extreme values of the five trials, with a rest interval of 10 s between jumps, were eliminated (best and worst), and the average of the three central values was used for the subsequent statistical analysis. The intraclass correlation coefficient (ICC) (95% CI) was 0.90 (95% CI, 0.88–0.92) for the SJ, 0.92 (95% CI, 0.90–0.94) for the CMJ and 0.91 (95% CI, 0.89–0.93) for the Abalakov jump.

#### 15-m Sprint Test

Sprint times were recorded outdoors on a BH sand pitch. Two types of sprint tests were performed, with (5 s of jumping and 5 s of skipping) and without pre-activation. Infrared beams were positioned at the sprint distance to be measured with a photoelectric cell (Muscle Lab. V7.18. Ergotest Technology; Langesund, Norway). Participants were allowed two practice trials performed at half speed after a thorough warm-up to familiarize them with the timing device. Two trials were completed, and the best-performance trial was used for the subsequent statistical analysis. A 3-min rest interval was permitted between 15-m trials. Two trials for each type of the sprint were completed. The ICC was 0.96 (95% CI, 0.94–0.98)

#### Throwing Speed Tests

All the throwing tests were performed on the sand (BH field). Players used their best technique to throw as fast as possible at a standard goal. Before the test, players followed a 10-min standardized warm-up using a standard BH ball (mass: 360 g, circumference: 17.5 cm). All throws were performed without the presence of a goalkeeper to avoid interference in the execution at maximum speed and were directed toward the center of the goal from the penalty line (6 m from the goal). Four types of overarm throws were performed: i) a standing (penalty) throw, ii) a 3-step running throw, iii) a jump throw, and iv) a 360º jump throw. Each participant completed three valid throws with each technique. The rest interval between throws was 10 s. The throwing speed was measured using Stalker-Sport-Radar^®,^ (Texas, USA). The radar device was positioned on a tripod behind the thrower (accurate to ± 0.1 km•h^−1^). The mean of the three attempts was used for subsequent statistical analyses. The ICC was 0.90 (95% CI, 0.88–0.92) for the standing (penalty) throw, 0.88 (95% CI, 0.86–0.90) for the 3-step running throw, 0.89 (95% CI, 0.87–0.91) for the jump throw and 0.87 (95% CI, 0.85–0.89) for the 360º jump throw.

#### Modified Course-Navette Endurance Test

The test was performed as previously described (Bago and Sáez de Villarreal, 2013). In this study, the test started at stage 10, at 12.52 km·h^−1^, using 20-m intervals. The rate of perceived exertion (RPE) data were collected immediately after the test, using the Borg 10-point scale (Borg et al., 1987). Afterward, the RPE value was multiplied by the total duration of the test in minutes (Foster et al., 2001), with the product representing in a single number the magnitude of internal effort in arbitrary units (AU). The total distance covered by the shuttles was recorded for analysis. The ICC was 0.91 (95% CI, 0.89–0.93). The maximum heart rate (HR_max_) (Polar S810, Kempele, Finland) and blood lactate concentration (Lactate Scout^®,^SensLab GmbH, Leipzig, Germany) were collected for baseline conditions, after the test and after 3 min of recovery. Participants were instructed not to perform any type of activity before the test, and to arrive at the laboratory 30 min in advance. After 20 min of rest, a first basal extraction was obtained with a Blood Lactate Scout analyzer (SensLab GmbH, Leipzig, Germany).

### Training Procedures

BH training was performed four days per week (M-W-Th-F) and the plyometric-sprint training program three days per week, during six weeks of intervention. The CG only performed regular BH training and the EG supplemented BH training with the plyometric-sprint training program. Each training session lasted 40 min (10 min of a standard warm-up, 25 min of plyometric-speed training and a 5-min cooldown). Players performed plyometric training including squat jumps, skipping, double-leg hops, double-leg speed hops, side hops, and alternative leg bounds. Sprint training consisted of a 15-m sprint after each repetition of jumps. Players rested one minute between each set. No special sprint, technical or plyometric training was performed by the CG. Training was performed on sand (the same as the competition), with participants using appropriated BH equipment. Plyometric + sprint training was performed before the start of the regular training session. Additionally, participants were encouraged to maintain their normal hydration levels, sleep, and dietary habits for the duration of the study. Table 2, describes the training program of the intervention.

**Table 2:** Training intervention.

	Week 1 Session 1–3	Week 2 Session 4–6	Week 3 Session 7–9	Week 4 Session 10–12	Week 5 Session 13–15	Week 6 Session 16–18
**Exercises**						
Squat jump + 15-m sprint	3 x 10	3 x 15	3 x 12	3 x 12	4 x 10	4 x 10
Skipping + 15-m sprint	3 x 10	3 x 15	3 x 12	3 x 12	4 x 10	4 x 10
Double-leg hops (40–60 cm) + 15-m sprint	3 (max)*	3 x Max	3 x Max	3 x Max	4 x Max	4 x Max
Double-leg speed hops + 15-m sprint	3 x 10	3 x 10	3 x 12	3 x 12	4 x 10	4 x 10
Side hop (2 m) + 15-m sprint	3 x 10	3 x 10	4 x 10	4 x 10	3 x 15	3 x 15
Alternated leg bounds + 15-m sprint	3 x 10 x 10 kg	3 x 10 x 10 kg	3 x 10 x 15 kg	3 x 10 x 15 kg	3 x 10 x 20 kg	3 x 10 x 20 kg

* : maximal effort, either as vertical height, minimal time contact, sprinting speed or a combination of these indexes

### Statistical Analyses

Descriptive statistics (mean ± SD) for all the considered variables were calculated. The Levene´s test was used to analyze the homogeneity of variance across groups and the Shapiro-Wilk test to evaluate the normality of distribution of the data. A mixed-design factorial analysis of variance with the contrast F of Snedecor assessed the training-related effects and differences between groups. Effect sizes (ESs) were calculated using Cohen’s *d* and statistical significance was accepted at an alpha level of *p* ≤ 0.05. The magnitude of the effect size statistics was considered trivial < 0.20; small, 0.20–0.50; moderate, 0.5–0.80; large, 0.8–1.30; and very large >1.30. The ES was reported in conjunction with the 95% confidence interval (CI) for all analyzed measures. The SPSS statistical package, version 24.0 was used (SPSS^®,^, Inc., Chicago, IL, USA).

## Results

At the pre-test, no significant differences between groups were observed in any variable (*p*> 0.05). After plyometric + sprint training, no significant changes were observed in anthropometrics (*p* > 0.05).

The experimental group presented significant increases (*p* ≤ 0.001) in the SJ (3.4 cm, 9.6% ± 1.7, ES = 0.70 ± 0.09), the CMJ (3.6 cm, 9.6% ± 1.7, ES = 0.73 ± 0.09) and the Abalakov jump (4.1 cm, 9.7% ± 1.8, ES = 0.84 ± 0.10) (Table 3). After the training intervention, significant differences (*p*< 0.05) were observed between the groups in the three vertical jump tests.

**Table 3:** Changes from pre- to post-intervention assessments in the selected performance variables for each group.

	Experimental group (EG)				Control group (CG)			
	(n = 12)				(n = 12)			
	Pre	Post	Δ%	ES	Pre	Post	Δ%	ES
Squat jump (cm)	35.53 ± 4.8	38.93 ± 5.1^ab^	9.56 ±1.7	0.70 ± 0.09	34.21 ± 3.4	35.03 ± 5.1	2.39 ± 0.6	0.24 ± 0.01
Countermovement jump (cm)	37.54 ± 4.9	41.15 ± 4.8 ^ab^	9.61 ± 1.7	0.73 ± 0.09	38.12 ± 5.2	39.05 ± 4.2	2.43 ± 0.6	0.17 ± 0.0
Abalakov jump (cm)	42.72 ± 5.1	46.87 ± 5.3 ^ab^	9.71 ± 1.8	0.84 ± 0.10	41.96 ± 4.8	42.87 ± 4.1	2.16 ± 0.5	0.18 ± 0.00
15-m sprint without pre-activation (s)	2.46 ± 0.11	2.38 ± 0.09 ^b^	3.25 ± 0.8	0.72 ± 0.09	2.44 ± 0.08	2.42 ± 0.12	0.81 ± 0.2	0.25 ±0.01
15-m sprint with pre-activation (s)	2.44 ± 0.11	2.37 ± 0.09 ^b^	2.84 ± 0.6	0.58 ± 0.05	2.45 ± 0.03	2.44 ± 0.16	0.40 ± 0.1	0.33 ± 0.02
Maximal heart rate (bpm)*	195.25 ± 9.4	196.83 ± 7.3	0.80 ± 0.2	0.16 ± 0.0	197.12 ± 10.2	198.26 ± 10.2	0.57 ± 0.1	0.11 ± 0.0
Lactate (mmol/L)*	8.73 ± 1.51	10.12 ± 1.64 ^ab^	15.92 ± 2.6	0.92 ± 0.12	9.18 ± 2.18	9.12 ± 2.61	0.65 ± 0.1	0.02 ± 0.0
Internal load (AU)*	50.57 ± 5.22	68.82 ± 6.12 ^ab^	36.08 ± 4.8	3.49 ± 0.40	46.12 ± 4.34	50.17 ± 5.78 ^b^	8.78 ± 1.6	0.93 ± 0.12
Modified Course-Navette endurance test (s)	337 ± 10.18	458 ± 11.19 ^ab^	35.90 ± 4.8	11.98 ± 1.2	328 ± 13.67	359 ± 15.34 ^b^	9.45 ± 1.7	2.26 ± 0.32
Penalty throw (km/h)	79.93 ± 3.66	81.41 ± 4.27	1.85 ± 0.4	0.40 ±0.04	81.32 ± 2.78	82.01 ± 2.46	0.84 ± 0.2	0.24 ± 0.01
3-step running throw (km/h)	83.75 ± 5.29	86.68 ± 5.94 ^b^	3.49 ± 0.8	0.55 ± 0.05	84.15 ± 4.11	84.76 ± 4.34	0.72 ± 0.2	0.14 ± 0.0
Jump throw (km/h)	78.00 ± 3.22	82.23 ± 3.58 ^ab^	5.42 ± 1.1	1.31 ± 0.17	78.62 ± 4.16	79.14 ± 4.25	0.66 ± 0.2	0.12 ± 0.0
360º jump throw (km/h)	70.48 ± 3.39	74.77 ± 4.03 ^ab^	6.08 ± 1.3	1.26 ± 0.17	70.89 ± 4.12	70.77 ± 4.21	−0.16 ± 0.0	0.02 ± 0.0

a Significant difference between groups (*p*< 0.05); ^b^ Significant difference between pre- and post-intervention (*p*< 0.05); ES: effect size; bpm: beats per minute; *: denotes values obtained after the completion of the Course-Navette test

The 15-m sprint without pre-activation (−0.08 s, 3.25% ± 0.8, ES = 0.72 ± 0.09) and with pre-activation (−0.07 s, 2.84% ± 0.6, ES = 0.58 ± 0.05) improved (*p*< 0.001) only in the experimental group. After the training intervention, no significant differences (*p*< 0.05) were observed between the two groups (Table 3).

The experimental group presented significant increases (*p*< 0.001) in the total time of the modified Course-Navette endurance test (121 s, 35.90% ± 4.8, ES = 11.98 ± 1.2), in the blood lactate concentration 3 min after the test (1.39 mmol/L, 15.92% ± 2.6, ES = 0.92 ± 0.12) and internal training loads (18.25 AU, 36.08% ± 4.8, ES = 3.49 ± 0.40). After the training intervention, significant differences were observed between both groups in the total time (*p* = 0.004), blood lactate concentration (*p* = 0.04) and internal training loads (*p*< 0.001) (Table 3).

Statistically significant increases (*p*< 0.001) were observed in the EG in the 3-steps running throw (2.93 km/h, 3.49% ± 0.8, ES = 0.55 ± 0.05), the jump throw (4.23 km/h, 5.42% ± 1.1, ES = 1.31 ± 0.17), and the 360º jump throw (4.29 km/h, 6.08% ± 1.3, ES = 1.26 ± 0.17). After the training intervention, significant differences were observed between the two groups in the jump throw (*p* = 0.02) and 360º jump throw tests (*p* = 0.005) (Table 3).

## Discussion

The benefits of PT and sprint training to physical fitness have been previously demonstrated after using hard surfaces (Aloui et al., 2022; Ramirez-Campillo et al., 2013, 2020), and soft surfaces (Ahmadi et al., 2021; Arazi et al., 2014; Binnie et al., 2014; Rajkumar and Devarishi, 2013). Our results, involving BH players, confirm the beneficial effects of sand-based (soft surface) PT, combined with sprint training, on athletes’ physical fitness.

As expected, vertical jump performance improved after the intervention. Of note, for the CMJ and the Abalakov jump, the magnitude of improvement was similar to the magnitude of improvement noted in the SJ. Although PT and sprint training exercises involved mainly SSC actions (in contrast to the pure-concentric action involved in the SJ test), PT on dry sand produced a significant increase in concentric power performance in the lower limbs when compared to harder surfaces (Arazi et al., 2014; Binnie et al., 2014; Impellizzeri et al., 2008; Miyama and Nosaka, 2004). Therefore, the added power requirement from jumping and sprinting on the sand may have increased maximal concentric power, and related physiological traits associated with the maximal-concentric strength (Wisloff et al., 2004). Indeed, our results are in line with those previously reported for male soccer players, where sand training was particularly effective to improve explosive strength in concentric-only actions (i.e., SJ) compared to a harder training surface (Impellizzeri et al., 2008). Similarly, PT over dry sand in volleyball players improved the rapid force of their lower limbs and their vertical jump height (Berriel et al., 2022; Rajkumar and Devarishi, 2013). However, the lack of physiological and biomechanical data collected in our intervention precludes us from providing a more robust mechanical approach to better understand the current findings (without undue speculation). Future studies may include such mechanistic measurements to provide further explanation regarding the role of sand-based interventions to particularly improve concentric-based muscle actions.

Regarding sprinting over dry sand, the intervention induced a significant within-group improvement (i.e., pre- vs. post-test improvement, *p* < 0.05), although no between-group difference was noted when compared to the control group (Table 3). The standard BH training and competition schedule of athletes involves frequent linear sprints. Therefore, athletes may have reached a high level of adaptation to linear sprint performance before the intervention, making it difficult to induce significant between-group differences in the short term, even considering that sand training may involve greater physiological adaptations via greater training stimuli (i.e., higher energy cost) (Binnie et al., 2014).

The progressive resistance test showed significant differences in such variables as total time of the test and blood lactate concentration after 3 min of recovery that could indicate that PT improves performance in the lower limbs, assuming a positive transfer to the BH players’ endurance capacity. In addition, maximum heart rate (HR) values were obtained, which would indicate the maximum-effort execution of the test by participants (Ahmaidi et al., 1992; Lemmink et al., 2004). The results showed a significant improvement (*p*< 0.05) in the time of the test in the EG compared to the pre-test. These data showed, in the first place, that based on the principles of adaptation and specificity, training on dry sand improved the endurance capacity since both groups improved their performance in the modified Course-Navette endurance test. However, the improvement in lower limb power has positive effects on endurance since this would favor displacement on dry sand, although efforts performed on this type of a surface cause greater energy expenditure (Binnie et al., 2014; Lejeune et al., 1998). Adapting to the environment involves improvements in performance factors such as, in this case, the ability to make a continuous incremental effort for a longer period. The HR_max_ average of the modified Course-Navette endurance test achieved during the pre-test was higher than the results obtained by male BH players (Pueo et al., 2017) and female BH players during a competition (Lara Cobos, 2011). It was also higher than values shown in other sports practiced on dry sand such as beach soccer (HR_max_ averages of 85.6% of the maximum with a 59.3% of the time of game above 90%) (Castellano and Casamichana, 2010). The ability of players to maintain the blood lactate concentration level during the matches will be essential to keep the game rhythm when the activity is carried out at maximum levels of the HR (Castellano and Casamichana, 2010; Lara Cobos, 2011). Blood lactate accumulated post-exercise is closely linked to the HR and the duration of the endurance test (Bago and Sáez de Villarreal, 2013). Significant changes were found in blood lactate concentration carried out three min after the completion of the resistance test in the EG. The concentration of lactate in the post-test in the EG agrees with another study found in the literature (Binnie et al., 2014). The results showed that PT increased the capacity to perform a progressive displacement effort with a change of direction on dry sand since participants were able to perform efforts for a longer time, at a higher speed and with a higher accumulated blood lactate concentration. This indicates that PT helps support greater metabolic stress corresponding to a higher internal load.

All handball throws in which the lower limbs participate (i.e., a 3-step running throw, a jump throw, a 360º jump throw) improved in the EG, similarly to the findings noted in handball players of different performance levels (Gorostiaga et al., 2006) and age (Ortega-Becerra et al., 2018). Those studies reported significant correlations between the power of lower limbs and the throwing speed (Gorostiaga et al., 2006; Ortega-Becerra et al., 2018). However, handball penalty throwing speed did not improve after the training intervention. This may be related to the reduced involvement of the lower limbs in the handball penalty throw. Indeed, 73% of the tangential velocity achieved by the ball in the handball penalty throw are due to the shoulder internal rotation and extension of the elbow, and the remaining 27% are attributable to the combined actions of other body segments (Dávila et al., 2011). In this sense, studies (Gorostiaga et al., 2006; van den Tillaar and Ettema, 2004) have concluded that there is no correlation between the power of the lower limbs and speed in a penalty throw in handball players. Therefore, the improvement of lower limb power, commonly occurring after PT (Sáez de Villarreal et al., 2010), particularly after sand-based PT, may explain the improvement in the speed of specific throws in BH, especially during particular conditions of dissipation of forces that take place on dry sand. Nonetheless, throwing is an open task. Therefore, future studies may determine how much of the improvement noted in this study may be transferred to competitive throwing, where players are faced with opponents and a constantly evolving game environment.

A possible limitation of the study could be the small sample size, but that often cannot be overcome studying elite athletes. Thus, it can be argued that greater improvement could have been achieved by increasing the number of players, exercises and weeks of treatment. Another factor that could contribute to the different outcomes between previous investigations concerning the associations between sprinting and throwing performance is the training and athlete’s background. Since we used BH athletes with extensive training and competition history, further gains are likely more difficult to achieve.

## Conclusions

Our findings indicate that compared to regular training, 6 weeks of preseason-combined plyometric and sprint training improved athletic performance and specific BH skills in high-level male BH players, including jumping, sprinting, and different types of throwing.
